# Adenoma arising from xanthoma in the transverse colon: a case report

**DOI:** 10.1093/gastro/goad013

**Published:** 2023-03-15

**Authors:** Yu Jin Jung, Jun Lee, Seong Jung Kim, Ran Hong

**Affiliations:** Department of Internal Medicine, College of Medicine, Chosun University, Gwangju, Republic of Korea; Department of Internal Medicine, College of Medicine, Chosun University, Gwangju, Republic of Korea; Department of Internal Medicine, College of Medicine, Chosun University, Gwangju, Republic of Korea; Department of Pathology, College of Medicine, Chosun University, Gwangju, Republic of Korea

## Introduction

A xanthoma is a benign lesion consisting of foamy cells characterized by a highly vacuolated cytoplasm. Such lesions can develop anywhere in the gastrointestinal tract but most often occur in the stomach, duodenum, or esophagus and very rarely in the colon [1]. Gastric xanthoma shows a high incidence in gastric cancer patients and is reportedly an independent predictor of gastric cancer [2]. Although adenomas associated with colorectal xanthoma were first reported in 1997, clear evidence supporting an association between xanthoma and colorectal adenoma or cancer is lacking [3]. Furthermore, the association between adenoma and lipid deposits is unproven, and although adenoma has been found adjacent to or around xanthoma, this is more likely coincidental.

Colorectal xanthomas are rare benign lesions with a small polypoid appearance and located mainly in the rectosigmoid area [4]. Here, we report a case of adenoma arising within a xanthoma treated with endoscopic submucosal dissection. The lesion reported in our case was distinguished from those in previous reports by its large size (>5 cm), atypical gross findings (laterally spreading), and unusual site (transverse colon). Accordingly, we report a case of xanthoma with an unusual gross finding along with a literature review.

## Case presentation

**Figure 1.**
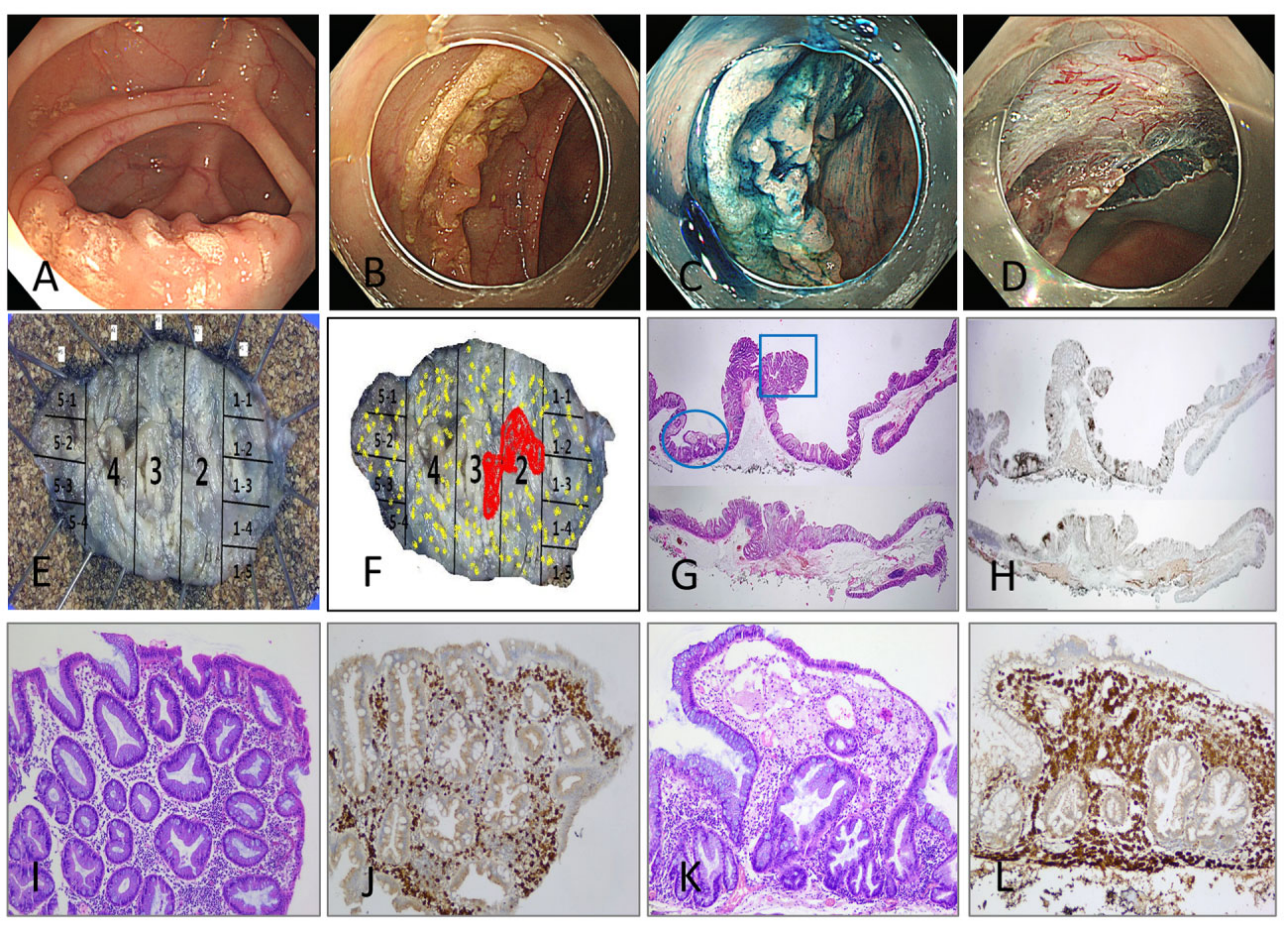
Endoscopic findings and histopathologic findings of the resected specimen. (A) Screening colonoscopy image shows a diffuse flat elevated lobular mucosal lesion resembling a starry sky with a yellow-colored patch in the transverse colon. (B) Colonoscopy with the distal attachment shows a 5.0 cm × 2.5 cm yellowish laterally spreading tumor in the transverse colon. (C) Chromo-endoscopy with indigo carmine shows the lesion clearly distinguished from the surrounding area. (D) The lesion was completely removed by endoscopic submucosal dissection using a dual knife without complications. (E) Gross specimen resected is divided into five sections. (F) Mapping of the resected specimen: low-grade tubular adenoma is visible at Segments 2 and 3 (red color) with xanthoma spreading diffusely over the entire segment (yellow color). (G) and (H) Histopathological examination of the endoscopic submucosal dissection specimen shows low-grade dysplasia in the background of CD68(+) and foamy histiocytic distribution (hematoxylin-eosin [H&E] and CD68 staining, respectively; both ×1.25). (I) and (J) High-power microscopic view of the circled area in (G) (H&E and CD68 staining, respectively; both ×10). (K) and (L) High-power microscopic view of the squared part in (G) (H&E and CD68 staining, respectively; both ×10).

A 71-year-old man with constipation underwent a screening colonoscopy in April 2017. The patient had no specific medical or family history. His serum triglyceride level was 43 mg/dL (normal, 42–168 mg/dL) and cholesterol level was 145 mg/dL (normal, 42–168 mg/dL); all other laboratory results were normal. Screening colonoscopy revealed a 5 cm × 2.5 cm yellow flat elevated mucosal lesion in the transverse colon. Endoscopic submucosal dissection was planned to enable an accurate diagnosis and definitive treatment. The procedure was performed using a dual knife (KD-650Q; Olympus, Tokyo, Japan) for circumferential mucosal cutting and the submucosal dissection. The lesion was completely removed without complications in 35 min. Histopathologic examination confirmed a 0.9 cm × 0.5 cm tubular adenoma with low-grade dysplasia in the background of an ∼5.0 cm × 2.5 cm–sized diffuse foamy histiocytic aggregate with clear resection margins. These foamy histiocytic cells were immunoreactive for CD68, suggesting colonic xanthoma (Figure 1). The patient was followed up without recurrence in surveillance colonoscopy performed after 1 year (April 2018) and 5 years (August 2022).

## Discussion

Lipid-laden macrophages, the main cells found in xanthomas, are mainly distributed in the lamina propria and expressing CD68 [5, 6]. Xanthomas can occur anywhere in the gastrointestinal tract, although the stomach is the most common site [1]. Colorectal xanthomas are extremely rare, with an unknown pathophysiology. Kim *et al*. [4] reviewed case reports and summarized 42 cases of colorectal xanthomas. Colorectal xanthomas were characterized as polypoid lesions on gross endoscopic examination ∼4.3 mm in diameter, red or white, and located in the rectosigmoid area.

Xanthomas are generally asymptomatic and incidentally discovered, and their association with adenomas or cancers is unclear. However, some cases of gastric cancer-related xanthoma have been reported, and a significant association between gastric cancer and xanthoma was confirmed in two retrospective studies [7, 8]. Moreover, Sekikawa *et al*. [2] reported in a multivariate analysis of a cohort study that the presence of gastric xanthoma was an independent predictor of early gastric cancer development. Although the exact mechanism of gastric cancer development in cases of xanthoma has not been proven, xanthoma is considered to be closely related to risk factors for precancerous lesions such as chronic atrophic gastritis, intestinal metaplasia, and *Helicobacter pylori* infection [9]. For example, phagocytosis in *H. pylori* can cause the transformation of macrophages into foamy cells; thus, *H. pylori* infection is considered a potential risk factor for xanthoma growth and development [9].

Colorectal xanthoma associated with adenoma was first reported by Boruchowicz *et al*. [3]. Since then, Kim *et al*. [4] reported that adenoma and adenocarcinomas were detected in ∼26% (11/42) and ∼5% (6/42) of xanthoma cases, respectively. As the proposed mechanism, researchers have postulated that diacylglycerol from foam cells is absorbed by colon cells and participates in cancer development by activating protein kinase C, which plays a role in signal transduction and growth regulation [3, 10]. However, thus far, it has been difficult to prove a direct association between adenomas and xanthomas, as the former can be adjacent to or around the latter. Our case had several important differences from previous xanthoma case reports. First, previous studies reported an average size of 4.3 mm and the largest reported case was only 1.5 cm, meaning that the xanthoma in our case is extremely large (∼5 cm). Second, regarding endoscopic gross findings, previous studies reported patches or small polyps, whereas our case showed a typical laterally spreading tumor. Third, the lesions of all cases previously reported were located in the rectosigmoid area, whereas those of our case developed in the transverse colon.

This report described a case of adenoma of atypical size, shape, and location (within a xanthoma) that was completely resected using endoscopic submucosal dissection. Although more case reports and histological associations should be demonstrated, we propose that endoscopic resection be considered in cases of xanthoma lesions with atypical size, shape, and location to enable their definitive diagnosis and treatment.

## Authors’ Contributions

Y.J.J. and J.L. designed the study; R.H., S.J.K., and Y.J.J. collected and analysed the data; Y.J.J and S.J.K. wrote the manuscript; J.L. and R.H. supervised coordination and the design of the study, and helped to draft the manuscript; S.J.K and J.L. did critical revision. All authors read and approved the final manuscript.

## References

[1] Gencosmanoglu R , Sen-Oran E, Kurtkaya-Yapicier O et al Xanthelasmas of the upper gastrointestinal tract. J Gastroenterol 2004;39:215–9. 15064997 10.1007/s00535-003-1288-3

[2] Sekikawa A , Fukui H, Sada R et al Gastric atrophy and xanthelasma are markers for predicting the development of early gastric cancer. J Gastroenterol 2016;51:35–42. 25904098 10.1007/s00535-015-1081-0

[3] Boruchowicz A , Rey C, Fontaine M et al Colonic xanthelasma due to glyceride accumulation associated with an adenoma. Am J Gastroenterol 1997;92:159–61. 8995960

[4] Kim SH , Kim HS, Choi YD et al A case of ascending colonic xanthoma presenting as a lateral spreading tumor. Intest Res 2014;12:162–5. 25349585 10.5217/ir.2014.12.2.162 PMC4204703

[5] Kaiserling E , Heinle H, Itabe H et al Lipid islands in human gastric mucosa: morphological and immunohistochemical findings. Gastroenterology 1996;110:369–74. 8566582 10.1053/gast.1996.v110.pm8566582

[6] Nakasono M , Hirokawa M, Muguruma N et al Colorectal xanthomas with polypoid lesion: report of 25 cases. APMIS 2004;112:3–10. 14961968 10.1111/j.1600-0463.2004.apm1120102.x

[7] Sekikawa A , Fukui H, Maruo T et al Gastric xanthelasma may be a warning sign for the presence of early gastric cancer. J Gastroenterol Hepatol 2014;29:951–6. 24372908 10.1111/jgh.12512

[8] Shibukawa N , Ouchi S, Wakamatsu S et al Gastric xanthoma is a predictive marker for metachronous and synchronous gastric cancer. World J Gastrointest Oncol 2017;9:327–32. 28868113 10.4251/wjgo.v9.i8.327 PMC5561044

[9] Moumin FA , Mohamed AA, Osman AA et al Gastric xanthoma associated with gastric cancer development: an updated review. Can J Gastroenterol Hepatol 2020;2020:3578927. 32149048 10.1155/2020/3578927 PMC7054765

[10] Reddy BS , Simi B, Engle A. Biochemical epidemiology of colon cancer: effect of types of dietary fiber on colonic diacylglycerols in women. Gastroenterology 1994;106:883–9. 8143994 10.1016/0016-5085(94)90746-3

